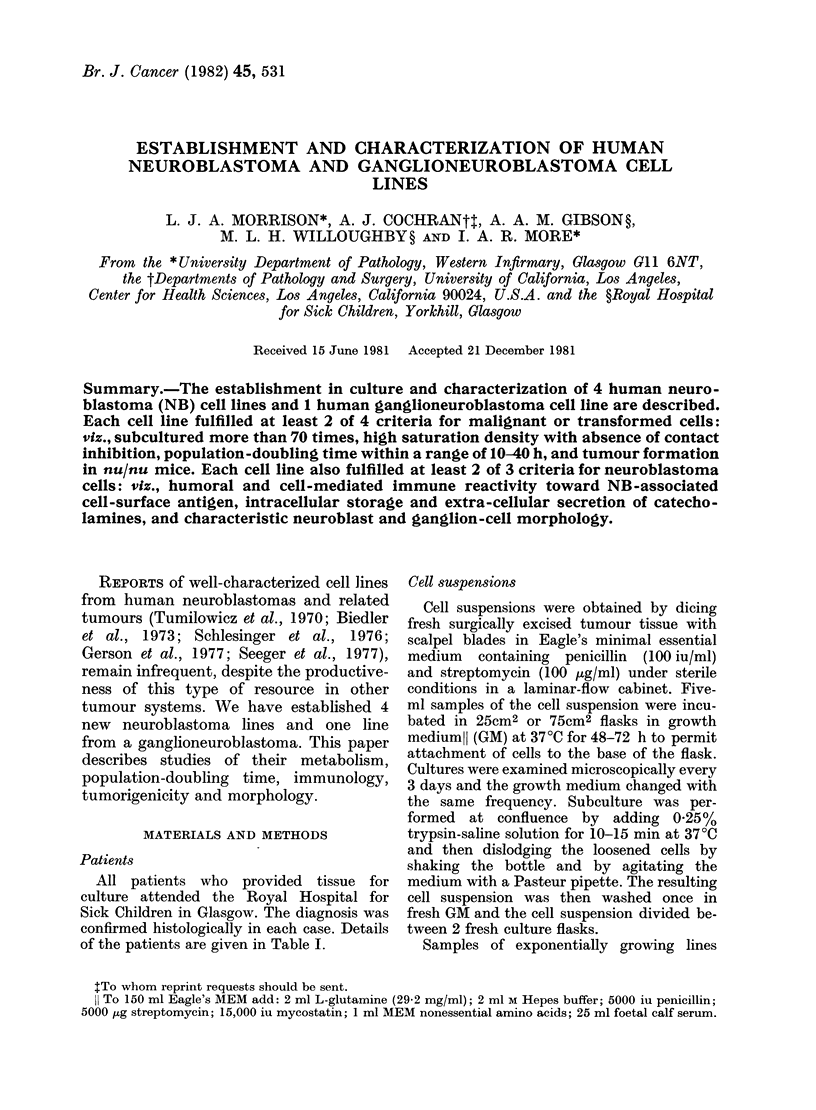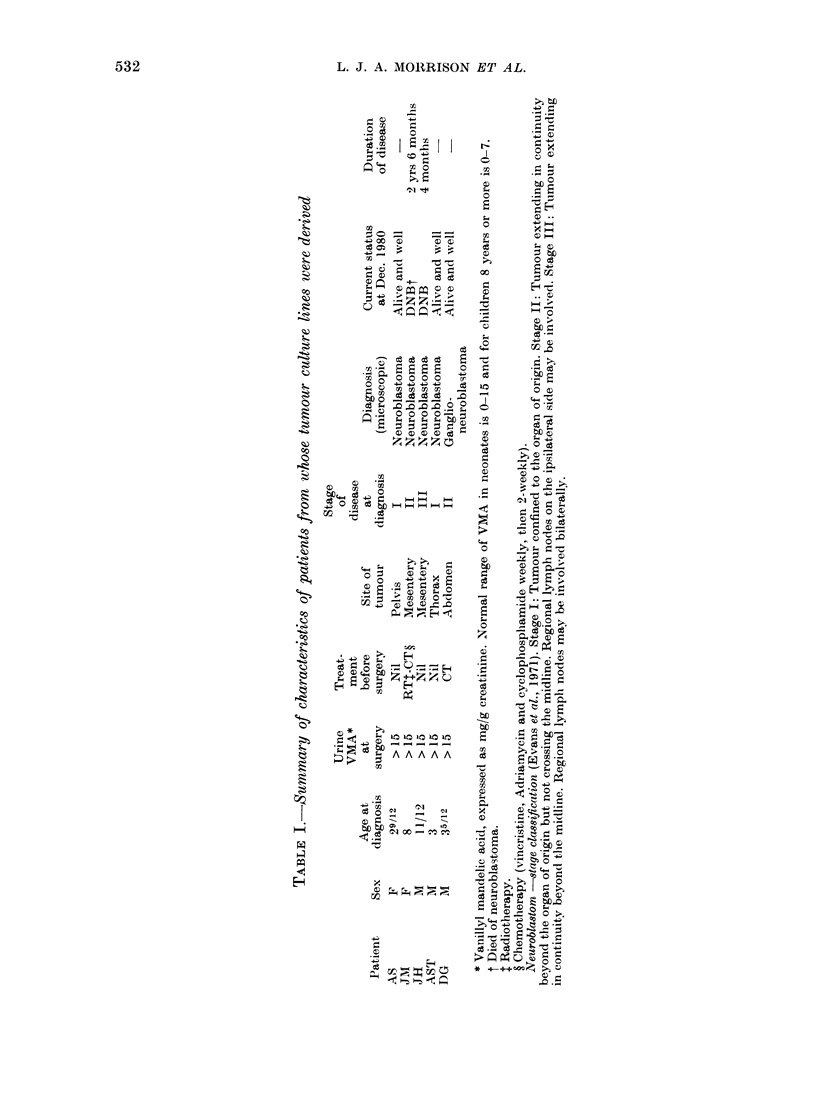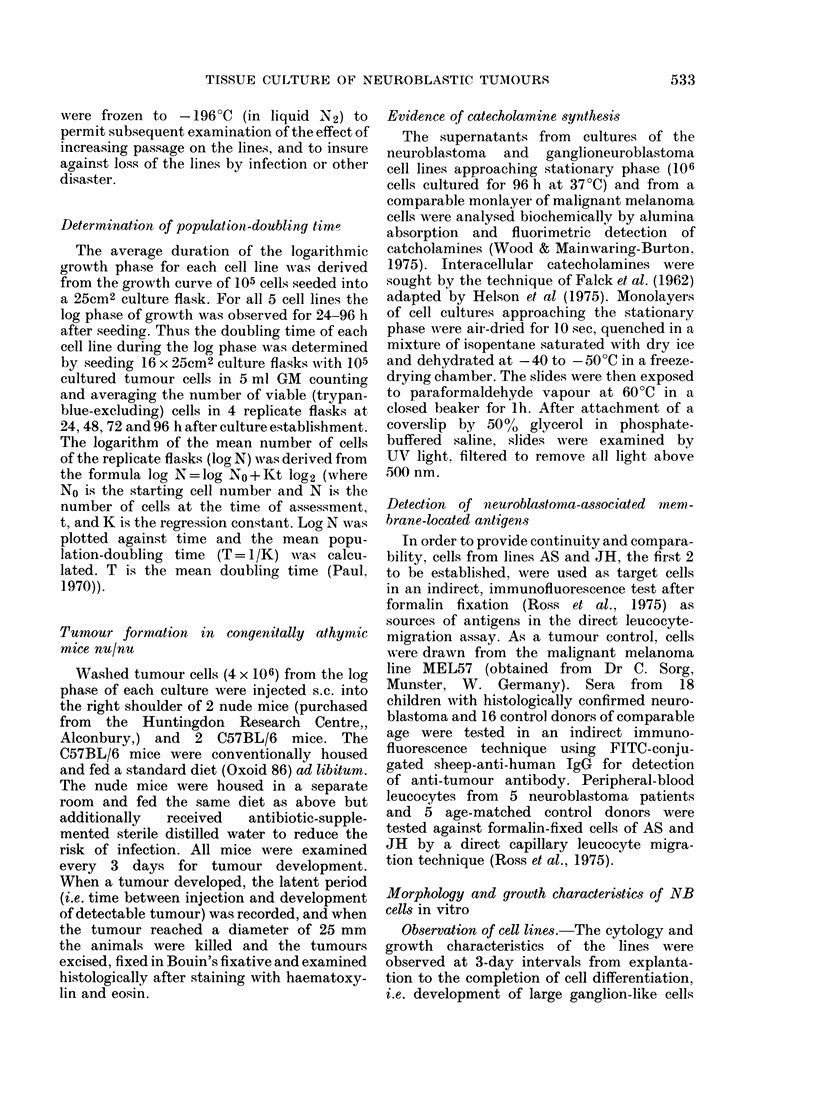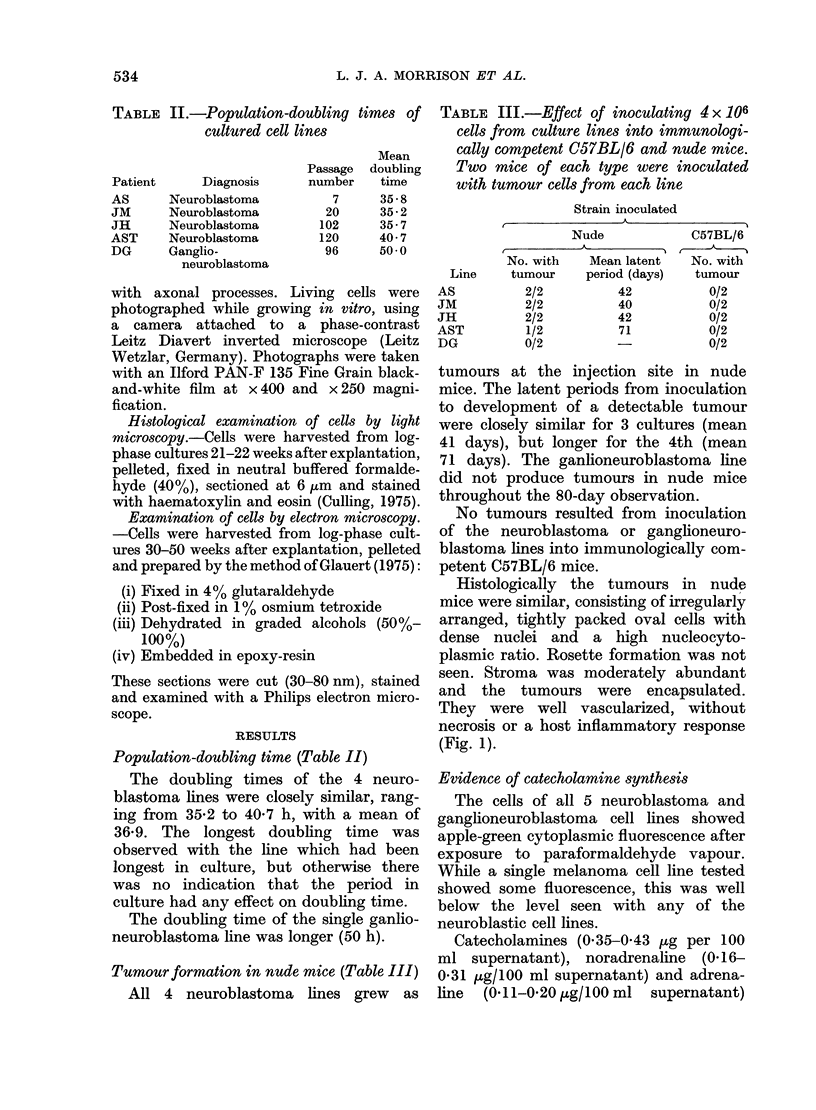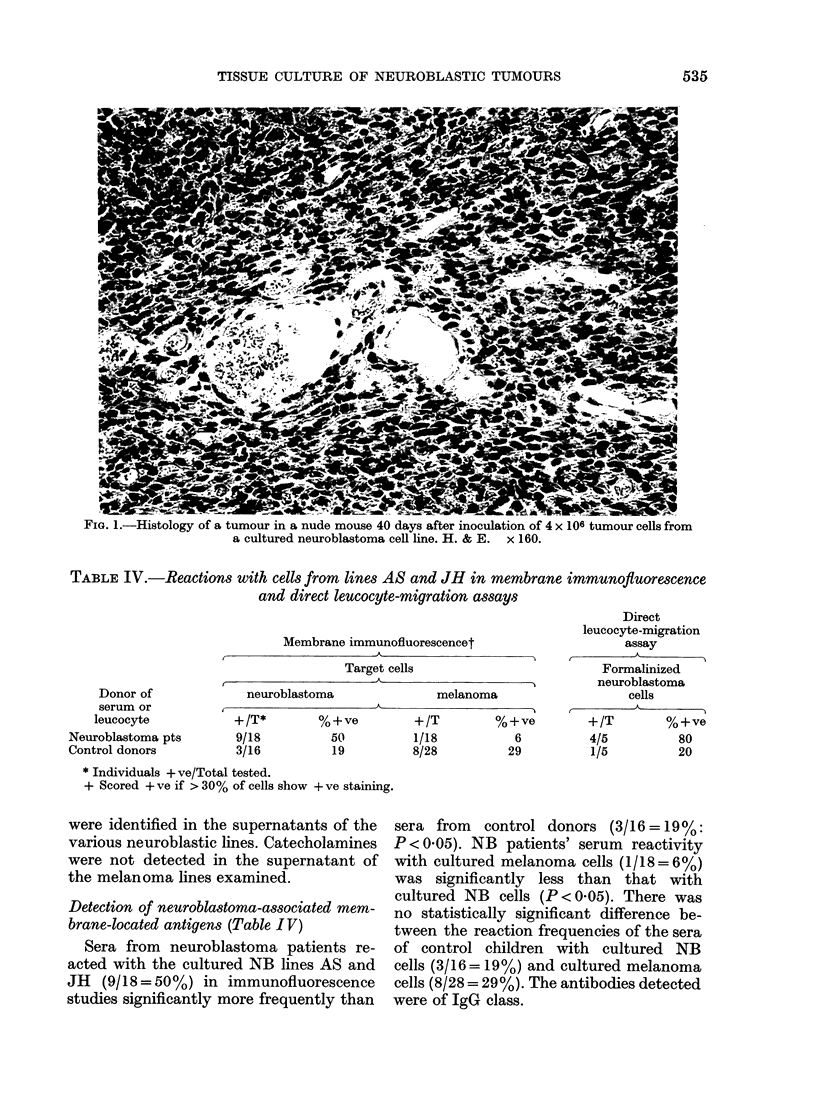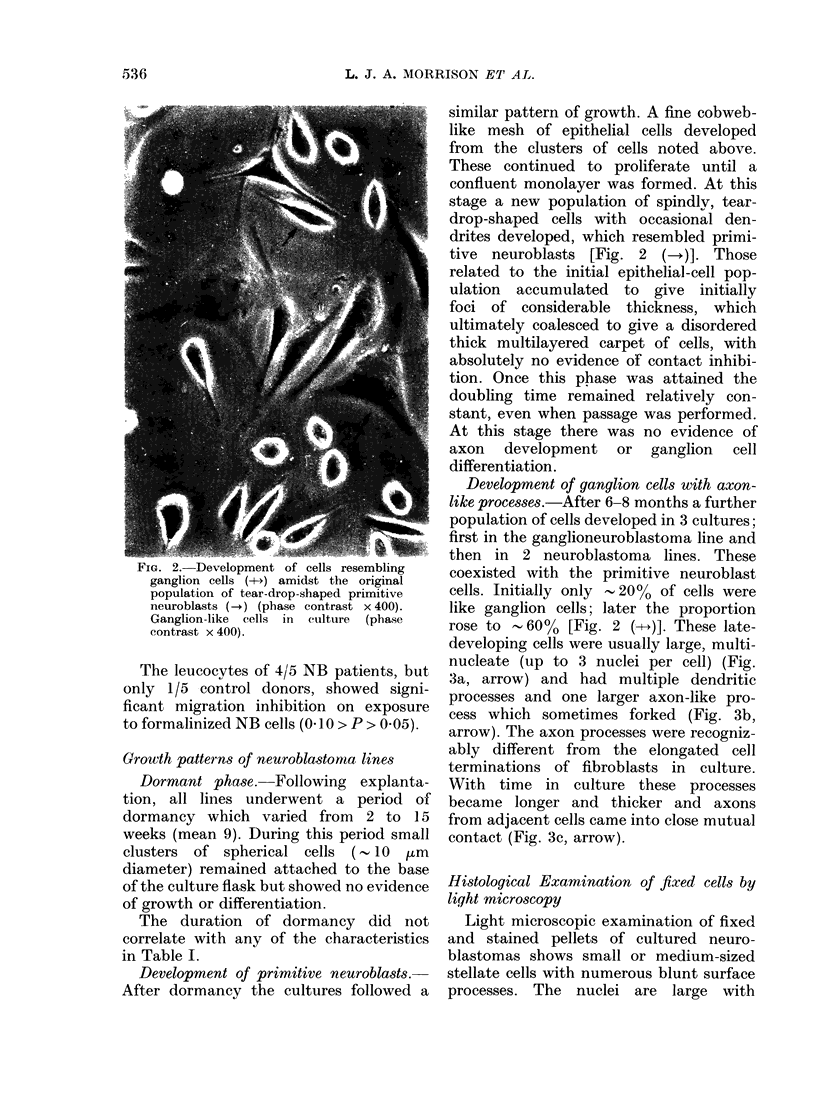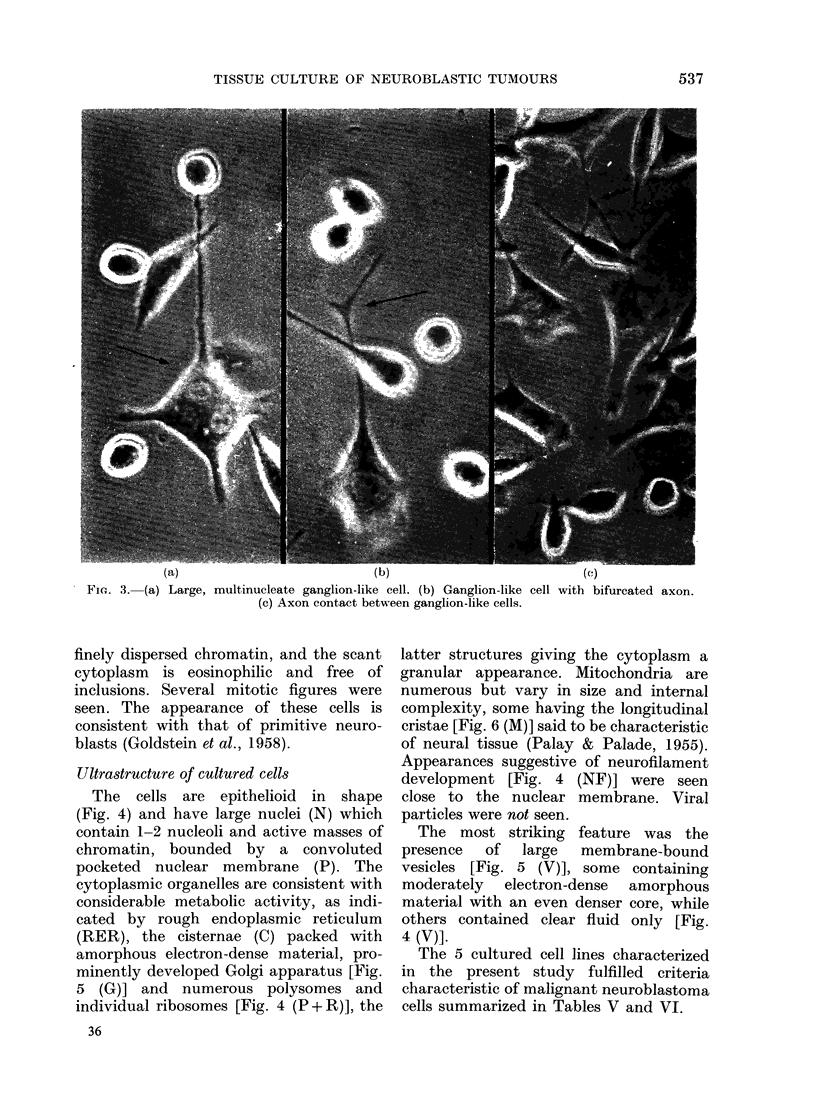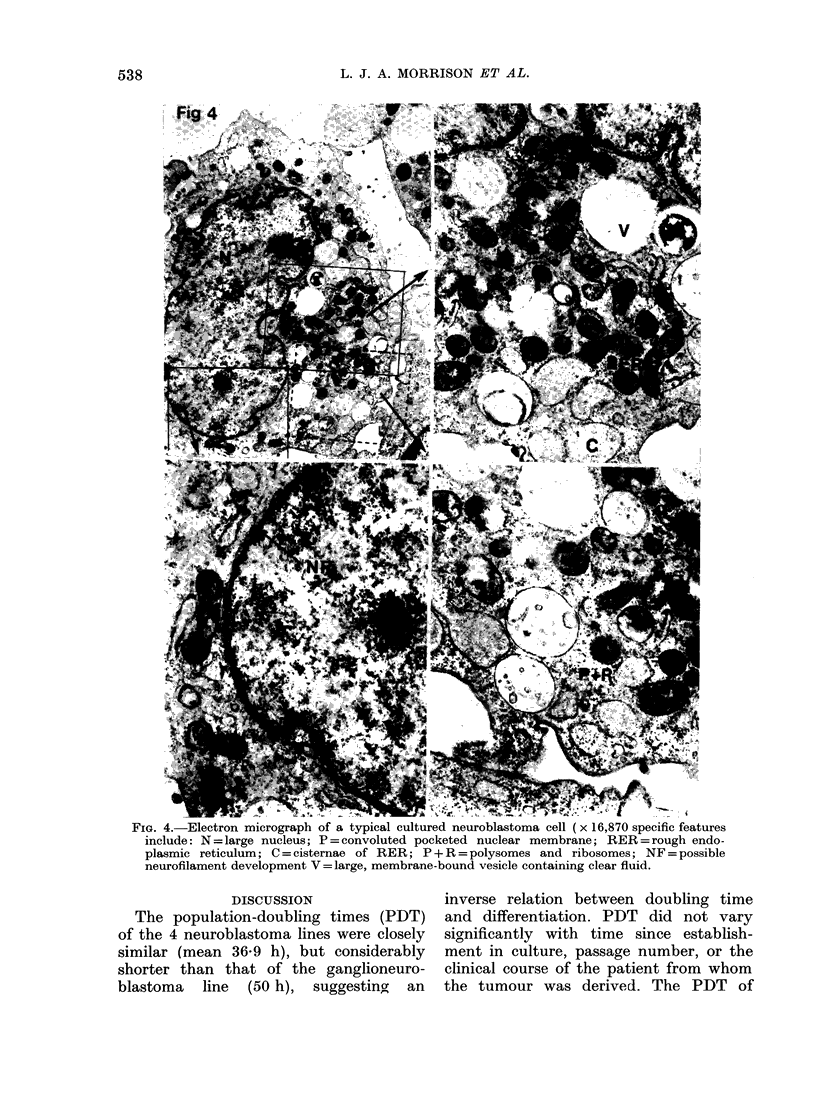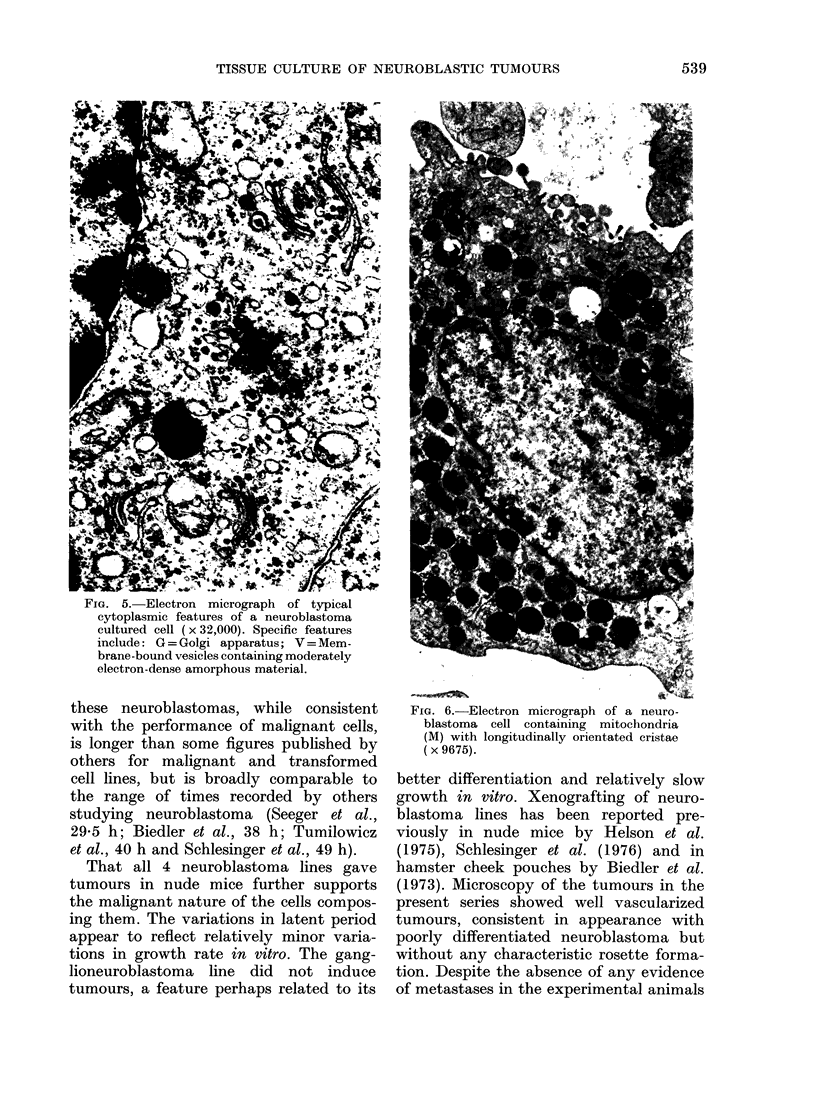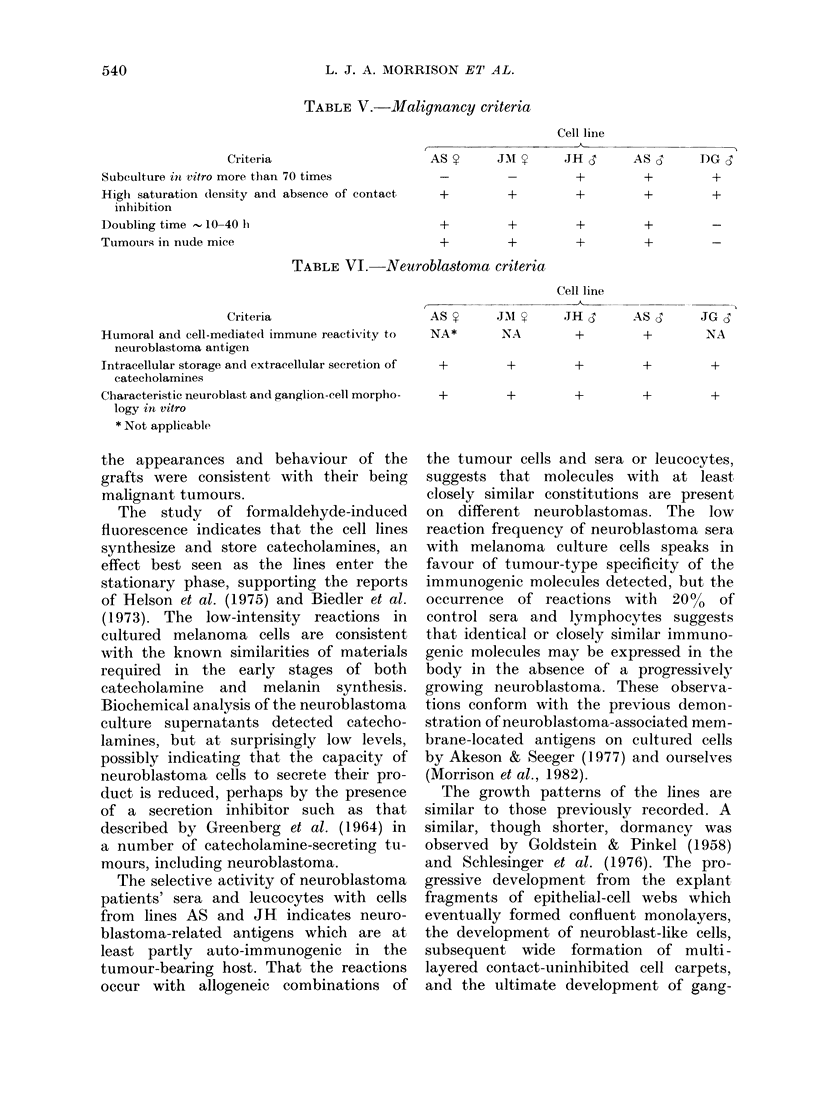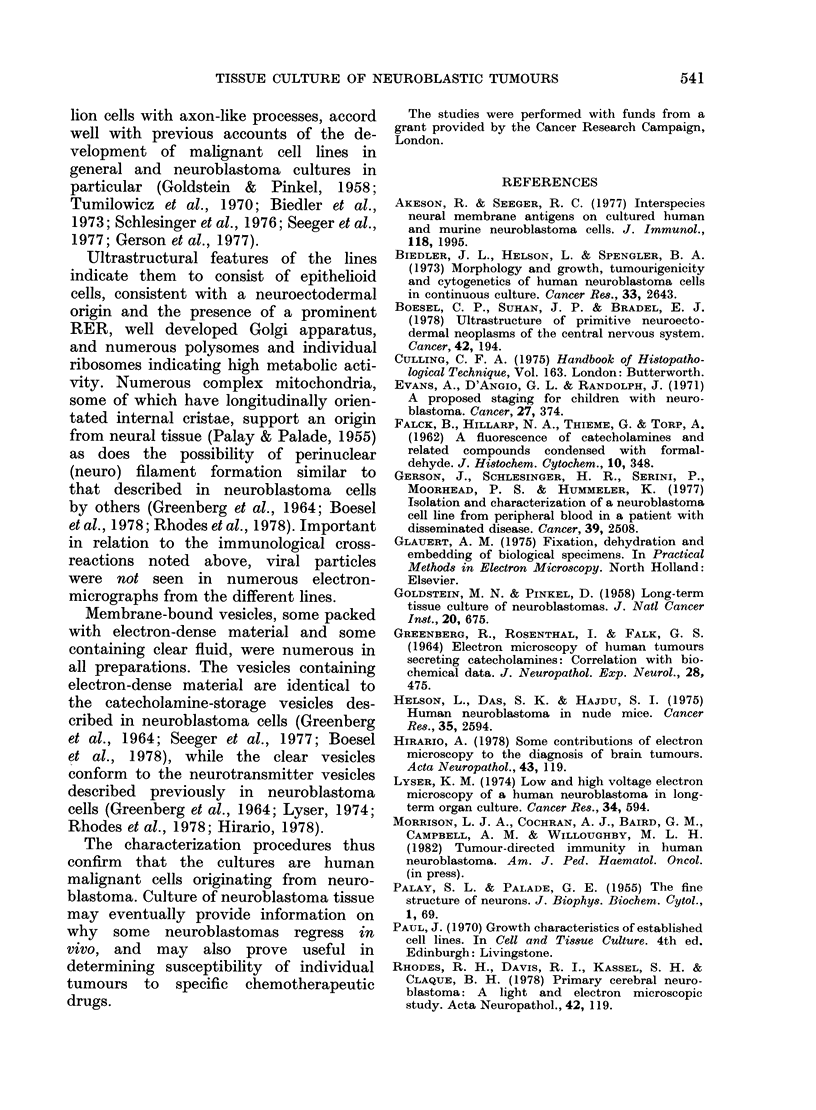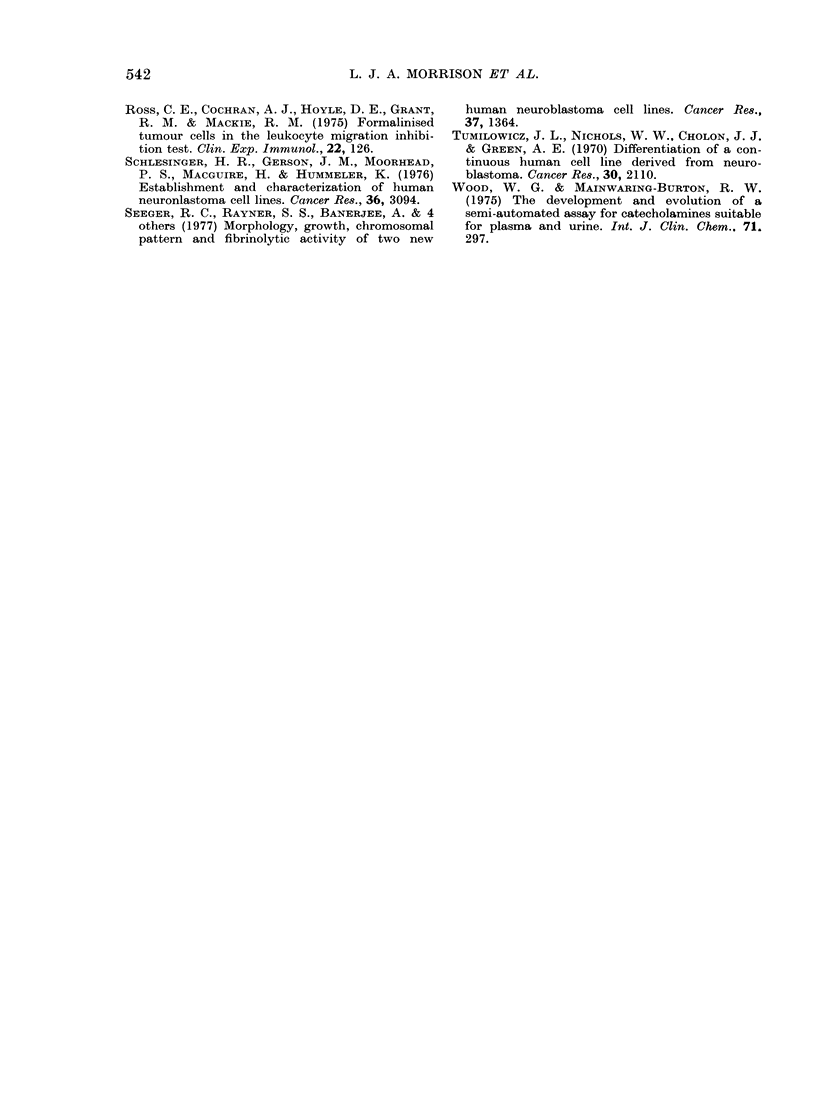# Establishment and characterization of human neuroblastoma and ganglioneuroblastoma cell lines.

**DOI:** 10.1038/bjc.1982.89

**Published:** 1982-04

**Authors:** L. J. Morrison, A. J. Cochran, A. A. Gibson, M. L. Willoughby, I. A. More

## Abstract

**Images:**


*Br. J. Cancer* (1982) **45**, 531

### ESTABLISHMENT AND CHARACTERIZATION OF HUMAN NEUROBLASTOMA AND GANGLIONEUROBLASTOMA CELL LINES

L. J. A. MORRISON*, A. J. COCHRAN†‡, A. A. M. GIBSON§, M. L. H. WILLOUGHBY§ AND I. A. R. MORE*

*From the *University Department of Pathology, Western Infirmary, Glasgow G11 6NT, the †Departments of Pathology and Surgery, University of California, Los Angeles, Center for Health Sciences, Los Angeles, California 90024, U.S.A. and the §Royal Hospital for Sick Children, Yorkhill, Glasgow*

Received 15 June 1981 Accepted 21 December 1981

**Summary.—The establishment in culture and characterization of 4 human neuroblastoma (NB) cell lines and 1 human ganglioneuroblastoma cell line are described. Each cell line fulfilled at least 2 of 4 criteria for malignant or transformed cells: *viz.*, subcultured more than 70 times, high saturation density with absence of contact inhibition, population-doubling time within a range of 10–40 h, and tumour formation in *nu/nu* mice. Each cell line also fulfilled at least 2 of 3 criteria for neuroblastoma cells: *viz.*, humoral and cell-mediated immune reactivity toward NB-associated cell-surface antigen, intracellular storage and extra-cellular secretion of catecholamines, and characteristic neuroblast and ganglion-cell morphology.**

REPORTS of well-characterized cell lines from human neuroblastomas and related tumours (Tumilowicz *et al.*, 1970; Biedler *et al.*, 1973; Schlesinger *et al.*, 1976; Gerson *et al.*, 1977; Seeger *et al.*, 1977), remain infrequent, despite the productiveness of this type of resource in other tumour systems. We have established 4 new neuroblastoma lines and one line from a ganglioneuroblastoma. This paper describes studies of their metabolism, population-doubling time, immunology, tumorigenicity and morphology.

### MATERIALS AND METHODS

### *Patients*

All patients who provided tissue for culture attended the Royal Hospital for Sick Children in Glasgow. The diagnosis was confirmed histologically in each case. Details of the patients are given in Table I.

### *Cell suspensions*

Cell suspensions were obtained by dicing fresh surgically excised tumour tissue with scalpel blades in Eagle's minimal essential medium containing penicillin (100 iu/ml) and streptomycin (100 $\mu$g/ml) under sterile conditions in a laminar-flow cabinet. Five-ml samples of the cell suspension were incubated in 25cm$^2$ or 75cm$^2$ flasks in growth medium‖ (GM) at 37°C for 48–72 h to permit attachment of cells to the base of the flask. Cultures were examined microscopically every 3 days and the growth medium changed with the same frequency. Subculture was performed at confluence by adding 0·25% trypsin-saline solution for 10–15 min at 37°C and then dislodging the loosened cells by shaking the bottle and by agitating the medium with a Pasteur pipette. The resulting cell suspension was then washed once in fresh GM and the cell suspension divided between 2 fresh culture flasks.

Samples of exponentially growing lines

‡To whom reprint requests should be sent.

‖ To 150 ml Eagle's MEM add: 2 ml L-glutamine (29·2 mg/ml); 2 ml M Hepes buffer; 5000 iu penicillin; 5000 $\mu$g streptomycin; 15,000 iu mycostatin; 1 ml MEM nonessential amino acids; 25 ml foetal calf serum.

TABLE I.—*Summary of characteristics of patients from whose tumour culture lines were derived*

| Patient | Sex | Age at diagnosis | Urine VMA* at surgery | Treatment before surgery | Site of tumour | Stage of disease at diagnosis | Diagnosis (microscopic) | Current status at Dec. 1980 | Duration of disease |
|---|---|---|---|---|---|---|---|---|---|
| AS | F | $2^{9/12}$ | > 15 | Nil | Pelvis | I | Neuroblastoma | Alive and well | — |
| JM | F | 8 | > 15 | RT‡-CT§ | Mesentery | II | Neuroblastoma | DNB† | 2 yrs 6 months |
| JH | M | 11/12 | > 15 | Nil | Mesentery | III | Neuroblastoma | DNB | 4 months |
| AST | M | 3 | > 15 | Nil | Thorax | I | Neuroblastoma | Alive and well | — |
| DG | M | $3^{5/12}$ | > 15 | CT | Abdomen | II | Ganglio-neuroblastoma | Alive and well | — |

* Vanillyl mandelic acid, expressed as mg/g creatinine. Normal range of VMA in neonates is 0–15 and for children 8 years or more is 0–7.
† Died of neuroblastoma.
‡ Radiotherapy.
§ Chemotherapy (vincristine, Adriamycin and cyclophosphamide weekly, then 2-weekly).

*Neuroblastom —stage classification* (Evans *et al.*, 1971). Stage I: Tumour confined to the organ of origin. Stage II: Tumour extending in continuity beyond the organ of origin but not crossing the midline. Regional lymph nodes on the ipsilateral side may be involved. Stage III: Tumour extending in continuity beyond the midline. Regional lymph nodes may be involved bilaterally.

were frozen to $-196°C$ (in liquid $N_2$) to permit subsequent examination of the effect of increasing passage on the lines, and to insure against loss of the lines by infection or other disaster.

*Determination of population-doubling time*

The average duration of the logarithmic growth phase for each cell line was derived from the growth curve of $10^5$ cells seeded into a $25cm^2$ culture flask. For all 5 cell lines the log phase of growth was observed for 24–96 h after seeding. Thus the doubling time of each cell line during the log phase was determined by seeding $16 \times 25cm^2$ culture flasks with $10^5$ cultured tumour cells in 5 ml GM counting and averaging the number of viable (trypan-blue-excluding) cells in 4 replicate flasks at 24, 48, 72 and 96 h after culture establishment. The logarithm of the mean number of cells of the replicate flasks (log N) was derived from the formula $\log N = \log N_0 + Kt \log_2$ (where $N_0$ is the starting cell number and N is the number of cells at the time of assessment, t, and K is the regression constant. Log N was plotted against time and the mean population-doubling time ($T = 1/K$) was calculated. T is the mean doubling time (Paul, 1970)).

*Tumour formation in congenitally athymic mice nu/nu*

Washed tumour cells ($4 \times 10^6$) from the log phase of each culture were injected s.c. into the right shoulder of 2 nude mice (purchased from the Huntingdon Research Centre,, Alconbury,) and 2 C57BL/6 mice. The C57BL/6 mice were conventionally housed and fed a standard diet (Oxoid 86) *ad libitum*. The nude mice were housed in a separate room and fed the same diet as above but additionally received antibiotic-supplemented sterile distilled water to reduce the risk of infection. All mice were examined every 3 days for tumour development. When a tumour developed, the latent period (*i.e.* time between injection and development of detectable tumour) was recorded, and when the tumour reached a diameter of 25 mm the animals were killed and the tumours excised, fixed in Bouin's fixative and examined histologically after staining with haematoxylin and eosin.

*Evidence of catecholamine synthesis*

The supernatants from cultures of the neuroblastoma and ganglioneuroblastoma cell lines approaching stationary phase ($10^6$ cells cultured for 96 h at 37°C) and from a comparable monlayer of malignant melanoma cells were analysed biochemically by alumina absorption and fluorimetric detection of catcholamines (Wood & Mainwaring-Burton, 1975). Interacellular catecholamines were sought by the technique of Falck *et al.* (1962) adapted by Helson *et al* (1975). Monolayers of cell cultures approaching the stationary phase were air-dried for 10 sec, quenched in a mixture of isopentane saturated with dry ice and dehydrated at $-40$ to $-50°C$ in a freeze-drying chamber. The slides were then exposed to paraformaldehyde vapour at 60°C in a closed beaker for 1h. After attachment of a coverslip by 50% glycerol in phosphate-buffered saline, slides were examined by UV light, filtered to remove all light above 500 nm.

*Detection of neuroblastoma-associated membrane-located antigens*

In order to provide continuity and comparability, cells from lines AS and JH, the first 2 to be established, were used as target cells in an indirect, immunofluorescence test after formalin fixation (Ross *et al.*, 1975) as sources of antigens in the direct leucocyte-migration assay. As a tumour control, cells were drawn from the malignant melanoma line MEL57 (obtained from Dr C. Sorg, Munster, W. Germany). Sera from 18 children with histologically confirmed neuroblastoma and 16 control donors of comparable age were tested in an indirect immunofluorescence technique using FITC-conjugated sheep-anti-human IgG for detection of anti-tumour antibody. Peripheral-blood leucocytes from 5 neuroblastoma patients and 5 age-matched control donors were tested against formalin-fixed cells of AS and JH by a direct capillary leucocyte migration technique (Ross *et al.*, 1975).

*Morphology and growth characteristics of NB cells* in vitro

*Observation of cell lines.*—The cytology and growth characteristics of the lines were observed at 3-day intervals from explantation to the completion of cell differentiation, *i.e.* development of large ganglion-like cells

TABLE II.—*Population-doubling times of cultured cell lines*

| Patient | Diagnosis | Passage number | Mean doubling time |
|---|---|---|---|
| AS | Neuroblastoma | 7 | 35·8 |
| JM | Neuroblastoma | 20 | 35·2 |
| JH | Neuroblastoma | 102 | 35·7 |
| AST | Neuroblastoma | 120 | 40·7 |
| DG | Ganglio-neuroblastoma | 96 | 50·0 |

with axonal processes. Living cells were photographed while growing *in vitro,* using a camera attached to a phase-contrast Leitz Diavert inverted microscope (Leitz Wetzlar, Germany). Photographs were taken with an Ilford PAN-F 135 Fine Grain black-and-white film at $\times 400$ and $\times 250$ magnification.

*Histological examination of cells by light microscopy.*—Cells were harvested from log-phase cultures 21–22 weeks after explantation, pelleted, fixed in neutral buffered formaldehyde (40%), sectioned at 6 $\mu$m and stained with haematoxylin and eosin (Culling, 1975).

*Examination of cells by electron microscopy.*—Cells were harvested from log-phase cultures 30–50 weeks after explantation, pelleted and prepared by the method of Glauert (1975):

(i) Fixed in 4% glutaraldehyde
(ii) Post-fixed in 1% osmium tetroxide
(iii) Dehydrated in graded alcohols (50%–100%)
(iv) Embedded in epoxy-resin

These sections were cut (30–80 nm), stained and examined with a Philips electron microscope.

### RESULTS

*Population-doubling time (Table II)*

The doubling times of the 4 neuroblastoma lines were closely similar, ranging from 35·2 to 40·7 h, with a mean of 36·9. The longest doubling time was observed with the line which had been longest in culture, but otherwise there was no indication that the period in culture had any effect on doubling time.

The doubling time of the single ganlioneuroblastoma line was longer (50 h).

*Tumour formation in nude mice (Table III)*

All 4 neuroblastoma lines grew as

TABLE III.—*Effect of inoculating* $4 \times 10^6$ *cells from culture lines into immunologically competent C57BL/6 and nude mice. Two mice of each type were inoculated with tumour cells from each line*

| | Strain inoculated | | |
|---|---|---|---|
| | Nude | | C57BL/6 |
| Line | No. with tumour | Mean latent period (days) | No. with tumour |
| AS | 2/2 | 42 | 0/2 |
| JM | 2/2 | 40 | 0/2 |
| JH | 2/2 | 42 | 0/2 |
| AST | 1/2 | 71 | 0/2 |
| DG | 0/2 | — | 0/2 |

tumours at the injection site in nude mice. The latent periods from inoculation to development of a detectable tumour were closely similar for 3 cultures (mean 41 days), but longer for the 4th (mean 71 days). The ganlioneuroblastoma line did not produce tumours in nude mice throughout the 80-day observation.

No tumours resulted from inoculation of the neuroblastoma or ganglioneuroblastoma lines into immunologically competent C57BL/6 mice.

Histologically the tumours in nude mice were similar, consisting of irregularly arranged, tightly packed oval cells with dense nuclei and a high nucleocytoplasmic ratio. Rosette formation was not seen. Stroma was moderately abundant and the tumours were encapsulated. They were well vascularized, without necrosis or a host inflammatory response (Fig. 1).

*Evidence of catecholamine synthesis*

The cells of all 5 neuroblastoma and ganglioneuroblastoma cell lines showed apple-green cytoplasmic fluorescence after exposure to paraformaldehyde vapour. While a single melanoma cell line tested showed some fluorescence, this was well below the level seen with any of the neuroblastic cell lines.

Catecholamines (0·35–0·43 $\mu$g per 100 ml supernatant), noradrenaline (0·16–0·31 $\mu$g/100 ml supernatant) and adrenaline (0·11–0·20 $\mu$g/100 ml supernatant)

FIG. 1.—Histology of a tumour in a nude mouse 40 days after inoculation of $4 \times 10^6$ tumour cells from a cultured neuroblastoma cell line. H. & E. ×160.

TABLE IV.—*Reactions with cells from lines AS and JH in membrane immunofluorescence and direct leucocyte-migration assays*

| Donor of serum or leucocyte | Membrane immunofluorescence† | | | | Direct leucocyte-migration assay | |
|---|---|---|---|---|---|---|
| | Target cells | | | | Formalinized neuroblastoma cells | |
| | neuroblastoma | | melanoma | | | |
| | +/T* | %+ve | +/T | %+ve | +/T | %+ve |
| Neuroblastoma pts | 9/18 | 50 | 1/18 | 6 | 4/5 | 80 |
| Control donors | 3/16 | 19 | 8/28 | 29 | 1/5 | 20 |

* Individuals +ve/Total tested.
+ Scored +ve if >30% of cells show +ve staining.

were identified in the supernatants of the various neuroblastic lines. Catecholamines were not detected in the supernatant of the melanoma lines examined.

*Detection of neuroblastoma-associated membrane-located antigens (Table IV)*

Sera from neuroblastoma patients reacted with the cultured NB lines AS and JH (9/18 = 50%) in immunofluorescence studies significantly more frequently than sera from control donors (3/16 = 19%: $P < 0{\cdot}05$). NB patients' serum reactivity with cultured melanoma cells (1/18 = 6%) was significantly less than that with cultured NB cells ($P < 0{\cdot}05$). There was no statistically significant difference between the reaction frequencies of the sera of control children with cultured NB cells (3/16 = 19%) and cultured melanoma cells (8/28 = 29%). The antibodies detected were of IgG class.

FIG. 2.—Development of cells resembling ganglion cells (+→) amidst the original population of tear-drop-shaped primitive neuroblasts (→) (phase contrast ×400). Ganglion-like cells in culture (phase contrast ×400).

The leucocytes of 4/5 NB patients, but only 1/5 control donors, showed significant migration inhibition on exposure to formalinized NB cells ($0{\cdot}10 > P > 0{\cdot}05$).

*Growth patterns of neuroblastoma lines*

*Dormant phase.*—Following explantation, all lines underwent a period of dormancy which varied from 2 to 15 weeks (mean 9). During this period small clusters of spherical cells ($\sim 10$ $\mu$m diameter) remained attached to the base of the culture flask but showed no evidence of growth or differentiation.

The duration of dormancy did not correlate with any of the characteristics in Table I.

*Development of primitive neuroblasts.*—After dormancy the cultures followed a similar pattern of growth. A fine cobweb-like mesh of epithelial cells developed from the clusters of cells noted above. These continued to proliferate until a confluent monolayer was formed. At this stage a new population of spindly, tear-drop-shaped cells with occasional dendrites developed, which resembled primitive neuroblasts [Fig. 2 (→)]. Those related to the initial epithelial-cell population accumulated to give initially foci of considerable thickness, which ultimately coalesced to give a disordered thick multilayered carpet of cells, with absolutely no evidence of contact inhibition. Once this phase was attained the doubling time remained relatively constant, even when passage was performed. At this stage there was no evidence of axon development or ganglion cell differentiation.

*Development of ganglion cells with axon-like processes.*—After 6–8 months a further population of cells developed in 3 cultures; first in the ganglioneuroblastoma line and then in 2 neuroblastoma lines. These coexisted with the primitive neuroblast cells. Initially only $\sim 20\%$ of cells were like ganglion cells; later the proportion rose to $\sim 60\%$ [Fig. 2 (+→)]. These late-developing cells were usually large, multinucleate (up to 3 nuclei per cell) (Fig. 3a, arrow) and had multiple dendritic processes and one larger axon-like process which sometimes forked (Fig. 3b, arrow). The axon processes were recognizably different from the elongated cell terminations of fibroblasts in culture. With time in culture these processes became longer and thicker and axons from adjacent cells came into close mutual contact (Fig. 3c, arrow).

*Histological Examination of fixed cells by light microscopy*

Light microscopic examination of fixed and stained pellets of cultured neuroblastomas shows small or medium-sized stellate cells with numerous blunt surface processes. The nuclei are large with

FIG. 3.—(a) Large, multinucleate ganglion-like cell. (b) Ganglion-like cell with bifurcated axon. (c) Axon contact between ganglion-like cells.

finely dispersed chromatin, and the scant cytoplasm is eosinophilic and free of inclusions. Several mitotic figures were seen. The appearance of these cells is consistent with that of primitive neuroblasts (Goldstein *et al.*, 1958).

*Ultrastructure of cultured cells*

The cells are epithelioid in shape (Fig. 4) and have large nuclei (N) which contain 1–2 nucleoli and active masses of chromatin, bounded by a convoluted pocketed nuclear membrane (P). The cytoplasmic organelles are consistent with considerable metabolic activity, as indicated by rough endoplasmic reticulum (RER), the cisternae (C) packed with amorphous electron-dense material, prominently developed Golgi apparatus [Fig. 5 (G)] and numerous polysomes and individual ribosomes [Fig. 4 (P + R)], the latter structures giving the cytoplasm a granular appearance. Mitochondria are numerous but vary in size and internal complexity, some having the longitudinal cristae [Fig. 6 (M)] said to be characteristic of neural tissue (Palay & Palade, 1955). Appearances suggestive of neurofilament development [Fig. 4 (NF)] were seen close to the nuclear membrane. Viral particles were *not* seen.

The most striking feature was the presence of large membrane-bound vesicles [Fig. 5 (V)], some containing moderately electron-dense amorphous material with an even denser core, while others contained clear fluid only [Fig. 4 (V)].

The 5 cultured cell lines characterized in the present study fulfilled criteria characteristic of malignant neuroblastoma cells summarized in Tables V and VI.

FIG. 4.—Electron micrograph of a typical cultured neuroblastoma cell ( × 16,870 specific features include: N = large nucleus; P = convoluted pocketed nuclear membrane; RER = rough endoplasmic reticulum; C = cisternae of RER; P + R = polysomes and ribosomes; NF = possible neurofilament development V = large, membrane-bound vesicle containing clear fluid.

### DISCUSSION

The population-doubling times (PDT) of the 4 neuroblastoma lines were closely similar (mean 36·9 h), but considerably shorter than that of the ganglioneuroblastoma line (50 h), suggesting an inverse relation between doubling time and differentiation. PDT did not vary significantly with time since establishment in culture, passage number, or the clinical course of the patient from whom the tumour was derived. The PDT of

FIG. 5.—Electron micrograph of typical cytoplasmic features of a neuroblastoma cultured cell (×32,000). Specific features include: G = Golgi apparatus; V = Membrane-bound vesicles containing moderately electron-dense amorphous material.

FIG. 6.—Electron micrograph of a neuroblastoma cell containing mitochondria (M) with longitudinally orientated cristae (×9675).

these neuroblastomas, while consistent with the performance of malignant cells, is longer than some figures published by others for malignant and transformed cell lines, but is broadly comparable to the range of times recorded by others studying neuroblastoma (Seeger *et al.*, 29·5 h; Biedler *et al.*, 38 h; Tumilowicz *et al.*, 40 h and Schlesinger *et al.*, 49 h).

That all 4 neuroblastoma lines gave tumours in nude mice further supports the malignant nature of the cells composing them. The variations in latent period appear to reflect relatively minor variations in growth rate *in vitro*. The ganglioneuroblastoma line did not induce tumours, a feature perhaps related to its better differentiation and relatively slow growth *in vitro*. Xenografting of neuroblastoma lines has been reported previously in nude mice by Helson *et al.* (1975), Schlesinger *et al.* (1976) and in hamster cheek pouches by Biedler *et al.* (1973). Microscopy of the tumours in the present series showed well vascularized tumours, consistent in appearance with poorly differentiated neuroblastoma but without any characteristic rosette formation. Despite the absence of any evidence of metastases in the experimental animals

TABLE V.—*Malignancy criteria*

| Criteria | Cell line AS ♀ | JM ♀ | JH ♂ | AS ♂ | DG ♂ |
|---|---|---|---|---|---|
| Subculture *in vitro* more than 70 times | − | − | + | + | + |
| High saturation density and absence of contact inhibition | + | + | + | + | + |
| Doubling time ∼10–40 h | + | + | + | + | − |
| Tumours in nude mice | + | + | + | + | − |

TABLE VI.—*Neuroblastoma criteria*

| Criteria | Cell line AS ♀ | JM ♀ | JH ♂ | AS ♂ | JG ♂ |
|---|---|---|---|---|---|
| Humoral and cell-mediated immune reactivity to neuroblastoma antigen | NA* | NA | + | + | NA |
| Intracellular storage and extracellular secretion of catecholamines | + | + | + | + | + |
| Characteristic neuroblast and ganglion-cell morphology *in vitro* | + | + | + | + | + |

* Not applicable the appearances and behaviour of the grafts were consistent with their being malignant tumours.

The study of formaldehyde-induced fluorescence indicates that the cell lines synthesize and store catecholamines, an effect best seen as the lines enter the stationary phase, supporting the reports of Helson *et al.* (1975) and Biedler *et al.* (1973). The low-intensity reactions in cultured melanoma cells are consistent with the known similarities of materials required in the early stages of both catecholamine and melanin synthesis. Biochemical analysis of the neuroblastoma culture supernatants detected catecholamines, but at surprisingly low levels, possibly indicating that the capacity of neuroblastoma cells to secrete their product is reduced, perhaps by the presence of a secretion inhibitor such as that described by Greenberg *et al.* (1964) in a number of catecholamine-secreting tumours, including neuroblastoma.

The selective activity of neuroblastoma patients' sera and leucocytes with cells from lines AS and JH indicates neuroblastoma-related antigens which are at least partly auto-immunogenic in the tumour-bearing host. That the reactions occur with allogeneic combinations of the tumour cells and sera or leucocytes, suggests that molecules with at least closely similar constitutions are present on different neuroblastomas. The low reaction frequency of neuroblastoma sera with melanoma culture cells speaks in favour of tumour-type specificity of the immunogenic molecules detected, but the occurrence of reactions with 20% of control sera and lymphocytes suggests that identical or closely similar immunogenic molecules may be expressed in the body in the absence of a progressively growing neuroblastoma. These observations conform with the previous demonstration of neuroblastoma-associated membrane-located antigens on cultured cells by Akeson & Seeger (1977) and ourselves (Morrison *et al.*, 1982).

The growth patterns of the lines are similar to those previously recorded. A similar, though shorter, dormancy was observed by Goldstein & Pinkel (1958) and Schlesinger *et al.* (1976). The progressive development from the explant fragments of epithelial-cell webs which eventually formed confluent monolayers, the development of neuroblast-like cells, subsequent wide formation of multilayered contact-uninhibited cell carpets, and the ultimate development of ganglion cells with axon-like processes, accord well with previous accounts of the development of malignant cell lines in general and neuroblastoma cultures in particular (Goldstein & Pinkel, 1958; Tumilowicz *et al.*, 1970; Biedler *et al.*, 1973; Schlesinger *et al.*, 1976; Seeger *et al.*, 1977; Gerson *et al.*, 1977).

Ultrastructural features of the lines indicate them to consist of epithelioid cells, consistent with a neuroectodermal origin and the presence of a prominent RER, well developed Golgi apparatus, and numerous polysomes and individual ribosomes indicating high metabolic activity. Numerous complex mitochondria, some of which have longitudinally orientated internal cristae, support an origin from neural tissue (Palay & Palade, 1955) as does the possibility of perinuclear (neuro) filament formation similar to that described in neuroblastoma cells by others (Greenberg *et al.*, 1964; Boesel *et al.*, 1978; Rhodes *et al.*, 1978). Important in relation to the immunological cross-reactions noted above, viral particles were *not* seen in numerous electron-micrographs from the different lines.

Membrane-bound vesicles, some packed with electron-dense material and some containing clear fluid, were numerous in all preparations. The vesicles containing electron-dense material are identical to the catecholamine-storage vesicles described in neuroblastoma cells (Greenberg *et al.*, 1964; Seeger *et al.*, 1977; Boesel *et al.*, 1978), while the clear vesicles conform to the neurotransmitter vesicles described previously in neuroblastoma cells (Greenberg *et al.*, 1964; Lyser, 1974; Rhodes *et al.*, 1978; Hirario, 1978).

The characterization procedures thus confirm that the cultures are human malignant cells originating from neuroblastoma. Culture of neuroblastoma tissue may eventually provide information on why some neuroblastomas regress *in vivo*, and may also prove useful in determining susceptibility of individual tumours to specific chemotherapeutic drugs.

The studies were performed with funds from a grant provided by the Cancer Research Campaign, London.

### REFERENCES

AKESON, R. & SEEGER, R. C. (1977) Interspecies neural membrane antigens on cultured human and murine neuroblastoma cells. *J. Immunol.*, **118**, 1995.

BIEDLER, J. L., HELSON, L. & SPENGLER, B. A. (1973) Morphology and growth, tumourigenicity and cytogenetics of human neuroblastoma cells in continuous culture. *Cancer Res.*, **33**, 2643.

BOESEL, C. P., SUHAN, J. P. & BRADEL, E. J. (1978) Ultrastructure of primitive neuroectodermal neoplasms of the central nervous system. *Cancer*, **42**, 194.

CULLING, C. F. A. (1975) *Handbook of Histopathological Technique*, Vol. 163. London: Butterworth.

EVANS, A., D'ANGIO, G. L. & RANDOLPH, J. (1971) A proposed staging for children with neuroblastoma. *Cancer*, **27**, 374.

FALCK, B., HILLARP, N. A., THIEME, G. & TORP, A. (1962) A fluorescence of catecholamines and related compounds condensed with formaldehyde. *J. Histochem. Cytochem.*, **10**, 348.

GERSON, J., SCHLESINGER, H. R., SERINI, P., MOORHEAD, P. S. & HUMMELER, K. (1977) Isolation and characterization of a neuroblastoma cell line from peripheral blood in a patient with disseminated disease. *Cancer*, **39**, 2508.

GLAUERT, A. M. (1975) Fixation, dehydration and embedding of biological specimens. In *Practical Methods in Electron Microscopy*. North Holland: Elsevier.

GOLDSTEIN, M. N. & PINKEL, D. (1958) Long-term tissue culture of neuroblastomas. *J. Natl Cancer Inst.*, **20**, 675.

GREENBERG, R., ROSENTHAL, I. & FALK, G. S. (1964) Electron microscopy of human tumours secreting catecholamines: Correlation with biochemical data. *J. Neuropathol. Exp. Neurol.*, **28**, 475.

HELSON, L., DAS, S. K. & HAJDU, S. I. (1975) Human neuroblastoma in nude mice. *Cancer Res.*, **35**, 2594.

HIRARIO, A. (1978) Some contributions of electron microscopy to the diagnosis of brain tumours. *Acta Neuropathol.*, **43**, 119.

LYSER, K. M. (1974) Low and high voltage electron microscopy of a human neuroblastoma in long-term organ culture. *Cancer Res.*, **34**, 594.

MORRISON, L. J. A., COCHRAN, A. J., BAIRD, G. M., CAMPBELL, A. M. & WILLOUGHBY, M. L. H. (1982) Tumour-directed immunity in human neuroblastoma. *Am. J. Ped. Haematol. Oncol.* (in press).

PALAY, S. L. & PALADE, G. E. (1955) The fine structure of neurons. *J. Biophys. Biochem. Cytol.*, **1**, 69.

PAUL, J. (1970) Growth characteristics of established cell lines. In *Cell and Tissue Culture*. 4th ed. Edinburgh: Livingstone.

RHODES, R. H., DAVIS, R. I., KASSEL, S. H. & CLAQUE, B. H. (1978) Primary cerebral neuroblastoma: A light and electron microscopic study. Acta Neuropathol., **42**, 119.

ROSS, C. E., COCHRAN, A. J., HOYLE, D. E., GRANT, R. M. & MACKIE, R. M. (1975) Formalinised tumour cells in the leukocyte migration inhibition test. *Clin. Exp. Immunol.*, **22**, 126.

SCHLESINGER, H. R., GERSON, J. M., MOORHEAD, P. S., MACGUIRE, H. & HUMMELER, K. (1976) Establishment and characterization of human neuronlastoma cell lines. *Cancer Res.*, **36**, 3094.

SEEGER, R. C., RAYNER, S. S., BANERJEE, A. & 4 others (1977) Morphology, growth, chromosomal pattern and fibrinolytic activity of two new human neuroblastoma cell lines. *Cancer Res.*, **37**, 1364.

TUMILOWICZ, J. L., NICHOLS, W. W., CHOLON, J. J. & GREEN, A. E. (1970) Differentiation of a continuous human cell line derived from neuroblastoma. *Cancer Res.*, **30**, 2110.

WOOD, W. G. & MAINWARING-BURTON, R. W. (1975) The development and evolution of a semi-automated assay for catecholamines suitable for plasma and urine. *Int. J. Clin. Chem.*, **71**, 297.